# Racial/ethnic and educational inequities in restrictive abortion policy variation and adverse birth outcomes in the United States

**DOI:** 10.1186/s12913-021-07165-x

**Published:** 2021-10-22

**Authors:** Sara K. Redd, Whitney S. Rice, Monica S. Aswani, Sarah Blake, Zoë Julian, Bisakha Sen, Martha Wingate, Kelli Stidham Hall

**Affiliations:** 1 Department of Health Policy and Management, Rollins School of Public Health, Emory University, 1518 Clifton Rd NE, Atlanta, GA 30322 USA; 2 Center for Reproductive Health Research in the Southeast (RISE), Emory University, 1518 Clifton Rd NE, Atlanta, GA 30322 USA; 3 Department of Behavioral, Social, and Health Education Sciences, Rollins School of Public Health, Emory University, 1518 Clifton Rd NE, Atlanta, GA 30322 USA; 4 Department of Health Services Administration, School of Health Professions, University of Alabama at Birmingham, 1719 9th Ave. S, Birmingham, AL 35233 USA; 5 Independent Clinician Scholar, Atlanta, GA 30322 USA; 6 Department of Health Care Organization and Policy, School of Public Health, University of Alabama at Birmingham, 1665 University Blvd, Birmingham, AL 35233 USA; 7 Department of Population and Family Health, Mailman School of Public Health, Columbia University, 722 West 168th St, New York, NY 10032 USA

**Keywords:** Abortion, Health policy, Health services research, Reproductive health services, Adverse birth outcomes, US state laws, Race/ethnicity, Education, Health disparities, Health inequities

## Abstract

**Background:**

To examine racial/ethnic and educational inequities in the relationship between state-level restrictive abortion policies and adverse birth outcomes from 2005 to 2015 in the United States.

**Methods:**

Using a state-level abortion restrictiveness index comprised of 18 restrictive abortion policies, we conducted a retrospective longitudinal analysis examining whether race/ethnicity and education level moderated the relationship between the restrictiveness index and individual-level probabilities of preterm birth (PTB) and low birthweight (LBW). Data were obtained from the 2005–2015 National Center for Health Statistics Period Linked Live Birth-Infant Death Files and analyzed with linear probability models adjusted for individual- and state-level characteristics and state and year fixed-effects.

**Results:**

Among 2,250,000 live births, 269,253 (12.0%) were PTBs and 182,960 (8.1%) were LBW. On average, states had approximately seven restrictive abortion policies enacted from 2005 to 2015. Black individuals experienced increased probability of PTB with additional exposure to restrictive abortion policies compared to non-Black individuals. Similarly, those with less than a college degree experienced increased probability of LBW with additional exposure to restrictive abortion policies compared to college graduates. For all analyses, inequities worsened as state environments grew increasingly restrictive.

**Conclusion:**

Findings demonstrate that Black individuals at all educational levels and those with fewer years of education disproportionately experienced adverse birth outcomes associated with restrictive abortion policies. Restrictive abortion policies may compound existing racial/ethnic, socioeconomic, and intersecting racial/ethnic and socioeconomic perinatal and infant health inequities.

**Supplementary Information:**

The online version contains supplementary material available at 10.1186/s12913-021-07165-x.

## Background

Over the last decade, states have enacted a variety of restrictive abortion policies at a rapidly increasing rate [1–3]. Since 2011, nearly 500 abortion restrictions have been enacted in states around the U. S [4], increasing the hostility of state environments towards abortion access. According to the Guttmacher Institute, over half of states are hostile to abortion rights, and 58% of women aged 15–44 reside in states considered hostile or very hostile to abortion services, meaning states had four or five (hostile), or six (very hostile) restrictive abortion policies in place [2]. Existing literature examining the implications of restrictive abortion policies have found associations between restrictive abortion policies and pregnant people planning to continue a pregnancy (versus seeking abortion) [5], decreased abortion rates [6–16], delayed abortions [9, 17–21], reduced provider availability [9, 10, 22–25], and increased financial and logistic barriers to care [26–32].

The increased implementation of state policies reducing access to abortion services limits the reproductive autonomy [33, 34] of those capable of pregnancy and has important implications for the health and wellbeing of these individuals and their children. As structural determinants of health [35] that shape who and via what means pregnant people can access abortion, pregnant people living in restrictive sociopolitical environments who are unable to access abortion services may end up continuing their pregnancy [5], resulting in adverse birth outcomes via a number of potential mechanisms. Because pregnancy and childbirth are inherently more dangerous and associated with more pregnancy-related morbidities and mortality than legal induced abortion, individuals unable to access abortion may be at increased risk for adverse birth outcomes [36–40]. Additionally, states with legal environments that restrict abortion access often lack supportive policies promoting the health and safety of pregnant people, their children, and their families – such as Medicaid expansion, expansive family/medical leave, and comprehensive sex education [41, 42] – which may result in reduced access to supportive services and thus contribute to adverse birth outcomes [33, 34, 42, 43]. Lastly, being unable to access desired medical care – in tandem with navigating structural barriers to obstetric care (e.g., lack of insurance coverage, documented shortages of obstetric providers [35]) and living in the historical and contemporary context of the United States – may increase psychosocial stress for the pregnant person, thus increasing their risk for adverse birth outcomes [44–47]. Indeed, research has linked restrictive abortion policies to increased rates of infant and maternal mortality, low birthweight, and child fatality and homicide deaths [47–54], and findings from our previous analysis suggest that increases in restrictive abortion policies were associated with increased probabilities of preterm birth and low birthweight in the Midwestern, Northeastern, and Western regions of the U.S. (Redd, et al. [2021]. Variation in Restrictive Abortion Policies and Adverse Birth Outcomes in the United States from 2005 to 2015, *in press*).

A substantial body of theory and literature has demonstrated the integral role that structural determinants, including health policy, play in influencing health and reinforcing health inequities [35, 55–58]. The United States has a rich history of enacting racist and classist policies at the federal and state level that explicitly or implicitly target, endanger, and even criminalize the fertility, sexuality, and reproduction of Black, Indigenous, immigrant, and lower-income populations [33–35, 59]. Indeed, restrictive abortion policies have become a critical macro-level factor shaping access to abortion care in the United States [42, 60] that inherently devalues the health and wellbeing of pregnant people. Research suggests that sociodemographic inequities [61–64] in abortion care are driven by structural factors [57] outside of an individual’s control, including decreased access to health care (including contraceptive services), provider availability, restrictions of insurance coverage of abortion, residential segregation, and economic disadvantage [61, 65, 66]. Given the persistent sociopolitical context surrounding reproductive policies in the U.S., and because people of color – particularly Black individuals – and those with lower socioeconomic statuses (SES) access abortion services at higher rates [63], restrictive abortion policies are likely to disproportionately affect these populations [67] and unduly influence health outcomes for pregnant people and their infants.

A handful of studies have demonstrated that restrictive abortion policies (or other policies governing reproductive rights) are associated with decreased access to abortion care for people of color and those with lower SES [67, 68] and increased risks of unintended teen births [69] and low birthweight among Black women [53]. Given the landscape of racial/ethnic and socioeconomic inequities in birth outcomes in the U.S. [70–86], policies restricting access to abortion have the potential to exacerbate adverse birth outcomes, as well as inequities in said outcomes, for people of color and those with lower SES. Furthermore, inequities in birth outcomes are intersectional [87]. Although higher education levels may be protective of adverse birth outcomes, numerous studies have demonstrated that, even among the highest educated groups, racial/ethnic inequities in birth outcomes persist, with Black individuals having the poorest health outcomes [88–93]. To our knowledge, few studies from the abortion policy literature base have explicitly examined how sociodemographic factors moderate the associations of restrictive abortion policies on adverse birth outcomes, resulting in a substantial conceptual gap in the field. As such, the objective of our paper was to assess whether the relationship between restrictive abortion policies and adverse birth outcomes was moderated by race/ethnicity and education level.

## Methods

In this analysis, we used linear probability modeling to determine if the relationship between state-level restrictive abortion policies and two individual-level adverse birth outcomes – preterm birth (PTB) and low birthweight (LBW) – varied for people of different racial/ethnic identities and education levels, from January 1, 2005 to December 31, 2015 in the United States.

### Data sources and measures

#### Outcomes

We defined PTBs as births occurring before 37 weeks gestation [94] and employed a binary 1/0 indicator for birth prior to 37 weeks versus 37 weeks or after. We classified births as LBW when less than 2500 g [94] and employed a binary 1/0 indicator for infant birthweight of less than 2500 g versus 2500 g or more. We obtained outcome data from the National Center for Health Statistics (NCHS) Period Linked Live Birth-Infant Death Files [95], which contain births occurring in all states and Washington, D.C., from 2005 through 2015.

#### Exposure: state-level restrictiveness index

Given the substantial increase in implementation of state-level policies restricting abortion across over the last decade, the abortion policy landscape is complex, highly varied, and difficult to evaluate quantitatively. In an effort to understand implications of the widely varying abortion policy environments, we sought to capture the restrictiveness of state environments towards abortion by creating an additive measure of enacted state restrictive abortion policies. We created and examined a composite state-level index of 18 restrictive abortion policies reducing abortion access and provision, using data from the National Association for the Repeal of Abortion Laws (NARAL) Pro-Choice America’s State Government Law and Policy databases [96] and Temple University’s Abortion Law Project [97]. We included the following restrictive abortion policies in our restrictiveness index: 1) abortion facility licensing requirements; 2) bans on insurance coverage of abortion for state employees; 3) bans on insurance coverage of abortion in health exchange plans; 4) bans on insurance coverage of abortion in all private insurance plans; 5) bans on public funding of abortion; 6) gestational age limits; 7) hospitalization requirements; 8) medication abortion administration only by licensed physician; 9) medication abortion administration in physical presence of patient; 10) mandatory counseling requirements; 11) mandatory ultrasound requirements; 12) mandatory waiting period requirements; 13) “partial-birth” abortion bans; 14) parental involvement laws; 15) abortion provision only by licensed physician; 16) physician hospital admitting privilege requirements; 17) provider refusal clauses; and 18) second physician requirements.

We coded policies as dichotomous beginning with the year the policy was enacted, with a “1” indicating that a policy was is in effect in a given state and year and a “0” indicating it was not. We included enjoined policies in the index, as policy enactment may still influence provider and patient behavior regardless of injunction status [98], until ruled unconstitutional. For instance, Alabama’s House Bill 57 [99] included a physician hospital admitting privilege requirement which was enacted in 2013, enjoined in 2013, and ruled unconstitutional in 2014. Using these inclusion criteria, we included this admitting privilege requirement in the index in 2013 and excluded it in 2014. We then summed the number of policies in each state and year – each policy counting separately – into a count variable. Thus, the final exposure measure was a state-level restrictiveness index aggregating the number of enacted restrictive abortion policies in a given state during a given year, with higher numbers representing greater restriction. The minimum number of policies a state may have in a given year was zero, and the maximum 18. Because policies enacted in a given year are not likely to affect infant outcomes until the subsequent year given the nine-month gestation period, we lagged the restrictiveness index by 1 year.

Lastly, as this index does not have a meaningful scale per se, we standardized the restrictiveness index in regression models in order to improve interpretation of this measure. Using this standardized measure, a value of zero represents the average number of restrictive abortion policies in the sample (approximately seven policies), and a one-unit change in the restrictiveness index represents a one standard deviation change (approximately four policies) in the index. Thus, we interpreted parameter estimates as changes given a one-standard deviation (SD), or four-policy, increase in the restrictiveness index. Additionally, we inspect predictive margins and average marginal effects of our analyses to probe how the relationship between race/ethnicity or education levels and our outcomes change across a range of values of restrictiveness index values.

#### Moderators: individual-level race/ethnicity and education

We investigate the potential moderating effect (i.e., interaction effects) of a birthing person’s race/ethnicity and education level, data which are derived from the NCHS dataset. Since these factors may lead to heterogeneity in the relationship between state-level abortion policies and adverse birth outcomes, simply controlling for them would not allow us to identify and understand inequities in birth outcomes along these dimensions. We operationalized a birthing person’s race/ethnicity in two ways. First, we employed a five-level categorical variable: American Indian/Alaska Native (AIAN) (non-Hispanic), Asian American/Pacific Islander (AAPI) (non-Hispanic), Black (non-Hispanic), Hispanic or Latinx, and White (non-Hispanic). Second, because the most substantial racial/ethnic inequities in birth outcomes are observed between Black and non-Black infants [75, 78, 80, 81, 83, 84, 86] and because Black individuals are disproportionately negatively impacted by structural determinants shaping access to health-promoting resources [35, 100], we employed a dichotomous 1/0 variable indicating whether a pregnant person identified as Black or non-Black (i.e., AIAN, AAPI, Hispanic or Latinx, and White). Regarding education level, we employed a four-level categorical variable: less than high school graduate, high school graduate or obtained GED, attended some college, and college graduate or beyond.

#### Covariates

We controlled for individual- and time-varying state-level demographic, economic, and political characteristics. In this analysis, “individual-level” refers to a unique parent-infant pair but is subsequently referred to as individual for simplicity. Individual-level covariate data came from the NCHS dataset and included birthing parent sociodemographic characteristics (i.e., age, marital status) and health risk factors (i.e., number of births and prenatal care visits, diabetes, chronic hypertension, pregnancy-associated hypertension, eclampsia) and infant characteristics (i.e., sex and plurality). State-level covariate data came from a variety of sources (e.g., American Community Survey, Bureau of Labor Statistics, Current Population Survey, Guttmacher Institute, National Center for Health Statistics, and the National Conference of State Legislatures) and included demographic (i.e., percentage of population that were at least high school graduates, married, and identified as people of color), economic (i.e., poverty, uninsured, and unemployment rates), and political and policy characteristics (i.e., state legislature composition, average monthly Temporary Assistance for Needy Families benefits [adjusted to 2010 dollars], Medicaid expansion, and Medicaid family planning expansion).

### Descriptive analysis

To identify inequities in adverse birth outcomes and exposure to restrictive abortion policies differed, we first conducted bivariate descriptive statistics using Pearson χ^2^ and one-way analysis of variance (ANOVA) tests.

### Main analysis

We investigate if the relationship between state-level restrictive abortion policies and individual-level probabilities of PTB and LBW differed by a person’s race/ethnicity and education level. We estimated the following general form of multivariate linear probability model [101] using state and year fixed-effects (FEs) [102]:

1 $$ {Y}_{ist}={\beta}_0+{\beta}_1{RI}_{s\left(t-1\right)}+{\beta}_2{M}_{ist}+{\beta}_3{RI}_{s\left(t-1\right)}\ast {M}_{ist}+{\beta}_4{X}_{1 ist}+{\beta}_5{X}_{2 st}+{\rho}_s+{\tau}_t+{\varepsilon}_{ist} $$

In Eq. 1, _*i*_ denotes an individual, _*s*_ denotes the state, and _*t*_ denotes the year. *Y*_*ist*_ represents the outcomes. *RI*_*s(t-1)*_ represents the standardized lagged restrictiveness index, or the number of restrictive abortion policies in effect in a state *s* during the previous year (*t-1*). *M*_*ist*_ represents the moderator of interest (i.e., race/ethnicity or education), and *RI*_s(t-1)_*M_*st*_ represents the interaction between the restrictiveness index and the moderating variable. This interaction term (β_3_) is our primary parameter of interest. *X*_*1ist*_ is the vector of individual-level covariates, and *X*_*2st*_ is the full vector of state-level covariates. *ρ*_*s*_ denotes state FEs accounting for time-invariant heterogeneity across states, while *τ*_*t*_ denotes year FEs accounting for national secular trends in the outcomes. *ε*_*ist*_ represents the error term.

We clustered standard errors at the state level in order to account for serial correlation of observations within states [103]. We opted to use linear probability models, rather than logit models, for improved efficiency and interpretability given the size of the sample and our inclusion of interaction terms and fixed effects. We, used Wald tests to examine the statistical significance of the interaction terms. For brevity, we only present and describe results from the interaction terms (including Wald tests) in each analysis (Table 2); results from full regression models are presented in Supplemental Tables 1, 2, and 3. Using estimates from the linear probability regression models, we provide predictive margins of both outcomes at various levels of the restrictiveness index (− 1 standard deviation [SD] to + 2 SD) for all racial/ethnic and educational subgroups in Table 3. Although not presented here, we further investigated the potential for moderation of the relationship between restrictive abortion policies and adverse birth outcomes by race/ethnicity and education via a three-way interaction (see Supplemental Tables 4, 5, 6 and Supplemental Figs. 4, 5, 6, and 7).

Additionally, we provide graphical interaction plots for models with significant interaction terms.^1^ The first set of plots display predictive margins of a given model, representing the predicted effect of the restrictiveness index on the probability of the outcome for individuals in each moderator category, controlling for all other covariates in the model. The second set of plots display average marginal effects for a given model, representing the predicted effect of the restrictiveness index on the probability of the outcome if values of the moderator are changed (e.g., comparing a specific group to the reference group), controlling for all other covariates in the model. For all plots, the x-axis (standardized lagged restrictiveness index) spans from − 2 SD to + 3 SD; this represents the approximate range of the standardized restrictiveness index in all states during the study period (actual values: − 1.71 SD to 2.53 SD).

Due to capacity issues caused by the large number of observations in the NCHS dataset (*N* = 44,992,972) and our modeling strategy, we drew a 5% state-year stratified random sample using proportional allocation, resulting in a final sample consisting of 2,250,000 births. To assess robustness of results, we repeated this sampling procedure twice with replacement; results were consistent across samples. We conducted all data management and analyses using SAS 9.4 and Stata/SE 16.0.

## Results

### Descriptive analysis

As shown in Table 1, across the study period, states had an average of seven restrictive abortion policies enacted, 12.0% of births were preterm, and 8.1% were low birthweight. Black individuals lived in states with the most enacted restrictive abortion policies (7.4), while AAPI and Hispanic/Latinx individuals lived in states with the fewest enacted restrictive abortion policies (4.6 and 5.3, respectively). In terms of adverse birth outcomes, Black individuals had the highest rates of PTB (17.2%) and LBW (13.5%), while AAPI and White individuals had the lowest rates of PTB (10.4 and 10.8%, respectively) and Hispanic/Latinx, White, and AIAN individuals had the lowest rates of LBW (7.0, 7.2, and 7.3%, respectively). Although the relationship between individual education levels and state restrictive abortion policies was statistically significant, there were no clear trends around exposure to restrictive abortion policies. However, rates of infant morbidity declined with increasing education levels; rates of PTB and LBW were highest among individuals with less than a high school education (13.8 and 9.1%) and lowest among college graduates (10.0 and 6.9%).

**Table 1:** Exposure to Restrictive Abortion Policies and Probability of Preterm Birth and Low Birthweight by Race/Ethnicity and Education Level: Period Linked Live Birth-Infant Death Files, 2005–2015 (*N* = 2,250,000)

	No. (%) or Mean ± SD		
	*Lagged Restrictiveness Index*	*Preterm Birth*	*Low Birthweight*
*Overall Sample*	7.0 ± 4.0	269,253 (12.0)	182,960 (8.1)
*Race/ethnicity (categorical)*	***	***	***
American Indian / Alaska Native (non-Hispanic) (*n* = 22,541)	7.0 ± 4.1	2985 (13.3)	1645 (7.3)
Asian American / Pacific Islander (non-Hispanic) (*n* = 136,921)	4.6 ± 3.7	14,141 (10.4)	11,260 (8.2)
Black (non-Hispanic) (*n* = 332,437)	7.4 ± 3.3	57,138 (17.2)	44,998 (13.5)
Hispanic/Latinx (*n* = 532,442)	5.3 ± 3.7	62,330 (11.8)	37,210 (7.0)
White (non-Hispanic) (*n* = 1,225,658)	7.1 ± 3.7	132,659 (10.8)	87,847 (7.2)
*Race/ethnicity (dichotomous)*	***	***	***
Black (non-Hispanic) (*n* = 332,437)	7.4 ± 3.3	57,138 (17.2)	44,998 (13.5)
Non-Black (*n* = 1,917,563)			
*Education*	***	***	***
Less than high school graduate (*n* = 404,170)	6.3 ± 3.7	55,627 (13.8)	36,719 (9.1)
High school graduate (*n* = 569,822)	6.7 ± 3.7	73,065 (12.9)	50,343 (8.8)
Some college (*n* = 568,116)	6.8 ± 3.8	67,046 (11.8)	45,138 (8.0)
College graduate (*n* = 602,021)	6.5 ± 3.7	60,162 (10.0)	41,195 (6.9)

*Note*: Results for categorical variables are unweighted numbers and proportions for each group. Results for continuous variables are means and standard deviations of each measure. *p*-values obtained from X^2^ analyses for categorical variables and one-way ANOVAs for continuous variables. *p*-values significant at ^***^ *p* < .001

### Linear probability regression models

Table 2 presents results from linear probability models examining the moderating effect of race/ethnicity (Sections A and B) and education (Section C) on the relationship between a state’s standardized restrictiveness index and the probability of PTB (column 1) and LBW (column 2).

**Table 2:** Linear Probability Models Examining Moderating Effects of Race/Ethnicity and Education Level on Relationship between Restrictiveness Index and Adverse Birth Outcomes: Period Linked Live Birth-Infant Death Files, 2005–2015 (*N* = 2,250,000)

		[1]	[2]
		*Preterm Birth* *(n = 2,058,512)*	*Low Birthweight* *(n = 2,061,512)*
***Section A:*** ***Race/ethnicity (categorical) x Restrictiveness Index (RI)***	**Interaction Term**		
	RI*AIAN (Non-Hispanic)	−0.00243 [− 0.00920, 0.00433]	0.0000550 [− 0.00460, 0.00471]
	RI*AAPI (Non-Hispanic)	− 0.00173 [− 0.00399, 0.000526]	0.000101 [− 0.00137, 0.00157]
	RI*Black (Non-Hispanic)	0.00247^*^ [0.000145, 0.00480]	0.00225 [− 0.000804, 0.00531]
	RI*Hispanic/Latinx	−0.00140 [− 0.00406, 0.00126]	−0.00171 [− 0.00371, 0.000281]
	RI*White (Non-Hispanic)	Ref.	Ref.
	*Wald Test of Interaction Term*	Χ^2^ = 1.69, *p* = 0.1681	Χ^2^ = 2.01, *p* = 0.1068
***Section B:*** ***Race/ethnicity (dichotomous) x Restrictiveness Index (RI)***	**Interaction Term**		
	RI*Black (Non-Hispanic)	0.00334^*^ [0.000809, 0.00587]	0.00292 [−0.000134, 0.00597]
	RI*Non-Black	Ref.	Ref.
	*Wald Test of Interaction Term*	Χ^2^ = 7.02, *p* = 0.0107	Χ^2^ = 3.69, *p* = 0.0606
***Section C:*** ***Education x Restrictiveness Index (RI)***	**Interaction Term**		
	RI*Less than HS	0.00190 [−0.000200, 0.00399]	0.00417^***^ [0.00178, 0.00656]
	RI*HS grad	0.00241^*^ [0.000446, 0.00436]	0.00360^***^ [0.00164, 0.00557]
	RI*Some college	0.00186^*^ [0.000327, 0.00340]	0.00153^**^ [0.000407, 0.00266]
	RI*College grad	Ref.	Ref.
	*Wald Test of Interaction Term*	Χ^2^ = 2.42, *p* = 0.0766	Χ^2^ = 5.17, *p* = 0.0034

*Note*: Results are marginal effects and 95% confidence intervals (CIs) from multivariate linear probability models estimating moderating effects of race/ethnicity and education level on the relationship between the standardized lagged restrictiveness index and the probability of preterm birth and low birthweight among all 50 states and Washington, D.C. Final sample size included people not missing any data on moderators, restrictiveness index, outcomes, and covariates. All models adjust for individual-level sociodemographic characteristics, state-level sociodemographic, economic, and political characteristics, and state and year fixed effects. Standard errors clustered at the state level. Wald test of interaction term tests if the interaction term as a whole is statistically significant. *p*-values significant at ^*^ *p* < .05, ^**^ *p* < .01, ^***^ *p* < .001

### Moderation by race/ethnicity

When employing the categorical race/ethnicity variable, we found that the relationship between the restrictiveness index and adverse birth outcomes did not vary by an individual’s racial/ethnic identity.^2^ However, when employing the dichotomous race/ethnicity variable (Black vs. non-Black), we observed a statistically significant interaction effect on PTB (χ^2^ = 7.02, *p* < 0.05), indicating that the relationship between a state’s restrictiveness index and the probability of PTB differed for Black and non-Black birthing people. A four-policy (one SD) increase in a state’s restrictiveness index among Black individuals increased the probability of PTB by 0.33 percentage points (95% CI: 0.008, 0.59; *p* < 0.05) compared to non-Black individuals, translating to a 2.8% increase in the probability of PTB among the sample (12.0 percentage points).

**Fig. 1:**
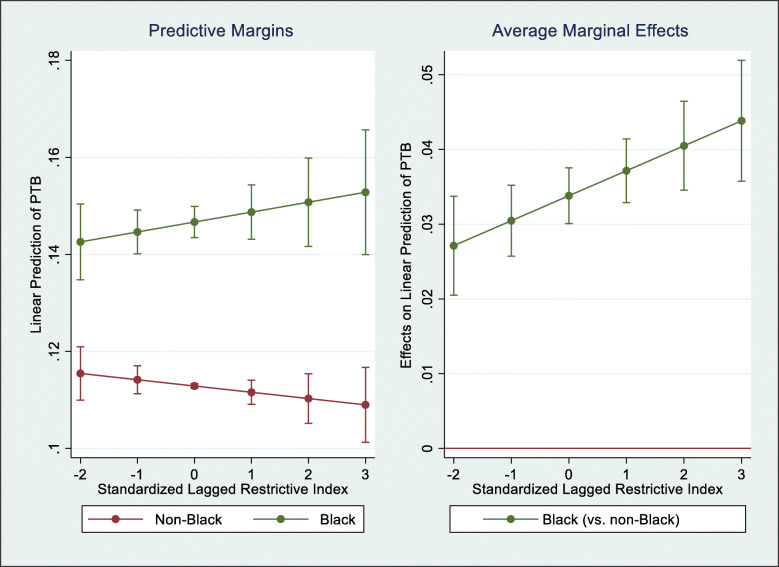
Predictive Margins and Average Marginal Effects of Racial/Ethnic (Dichotomous) Inequities in Relationship between Restrictiveness Index and Preterm Birth. Note: Results are predictive margins and average marginal effects from multivariate linear probability models estimating the moderating effect of race/ethnicity (dichotomous) on the relationship between the standardized lagged restrictiveness index and the probability of preterm birth among all 50 states and Washington, D.C. Final sample size included people not missing any data on race/ethnicity, restrictiveness index, preterm birth, and covariates. All models adjust for individual-level sociodemographic characteristics, state-level sociodemographic, economic, and political characteristics, and state and year fixed effects. Standard errors clustered at the state level

These effects are presented as predictive margins in Table 3 and the left panel of Fig. 1, where we see the inequity in predicted values of PTB for Black and non-Black individuals, which worsen as states enact more restrictive abortion policies. In the least restrictive states (− 1 SD), the predicted PTB values were 14.5% for Black birthing people and 11.1% for non-Black birthing people, a difference of 3.4 percentage points. In the most restrictive states (+ 2 SD), the predicted value of PTB grew to 15.1% for Black birthing people and declined to 11% for non-Black birthing people, a difference of 4.1 percentage points. The right panel of Fig. 1 presents the average marginal effects of being Black (versus non-Black) on the relationship between the restrictiveness index and the predicted probability of the PTB. As with the predictive margins, we see how the effect of being Black (versus being non-Black) on the predicted probability of PTB increased as states grew increasingly restrictive.

**Table 3:** Predictive Margins of Preterm Birth and Low Birthweight from Linear Probability Models Examining Moderating Effects of Race/Ethnicity or Education Level on Relationship between Restrictiveness Index and Adverse Birth Outcomes

	Preterm Birth				Low Birthweight			
	*−1 SD*	*0 SD*	*+ 1 SD*	*+ 2 SD*	*−1 SD*	*0 SD*	*+ 1 SD*	*+ 2 SD*
***Race/ethnicity (categorical) x Restrictiveness Index (RI)***								
AIAN	11.6	11.2	10.9	10.6	6.3	6.0	5.8	5.6
AAPI	11.9	11.7	11.4	11.2	9.5	9.2	9.0	8.8
Black	14.5	14.7	14.8	15.0	11.4	11.4	11.4	11.5
Hispanic or Latinx	11.7	11.4	11.2	11.0	7.2	6.8	6.4	6.0
White	11.2	11.1	11.1	11.0	7.5	7.3	7.1	6.9
***Race/ethnicity (dichotomous) x Restrictiveness Index (RI)***								
Black	14.5	14.7	14.9	15.1	11.5	11.5	11.5	11.5
Non-Black	11.4	11.3	11.1	11.0	7.6	7.3	7.0	6.8
***Education Level x Restrictiveness Index (RI)***								
LT HS grad	12.6	12.5	12.5	12.4	8.7	8.7	8.7	8.7
HS grad	12.3	12.3	12.3	12.3	8.5	8.5	8.4	8.3
Some college	12.0	11.9	11.9	11.8	8.2	7.9	7.6	7.3
College grad	10.9	10.7	10.4	10.2	7.4	6.9	6.5	6.1

*Note*: Results are predictive margins of preterm birth and low birthweight for all racial/ethnic and education level subgroups at −1 standard deviation (SD), 0 SD, + 1 SD, and + 2 SD of the lagged restrictiveness index. Predictive margin estimates were produced from multivariate linear probability models estimating moderating effects of race/ethnicity or education level on the relationship between the standardized lagged restrictiveness index and the probability of preterm birth and low birthweight among all 50 states and Washington, D.C. Final sample size included people not missing any data on race/ethnicity, education level, restrictiveness index, outcomes, and covariates. All models adjust for individual-level sociodemographic characteristics, state-level sociodemographic, economic, and political characteristics, and state and year fixed effects. Standard errors clustered at the state level

### Moderation by education level

When examining moderation by education level, we observe a statistically significant interaction effect on LBW (χ^2^ = 5.17, *p* < 0.01), indicating that the relationship between a state’s restrictiveness index and the probability of LBW differed by an individual’s level of education. A four-policy (one SD) increase in a state’s restrictiveness index among those with less than a high school education increased the probability of LBW by 0.42 percentage points (95% CI: 0.18, 0.66; *p* < 0.001) compared to college graduates, a 5.1% increase in the probability of LBW among the sample (8.3 percentage points). A four-policy increase in a state’s restrictiveness index among high school graduates increased the probability of LBW by 0.36 percentage points (95% CI: 0.16, 0.56; *p* < 0.001) compared to college graduates, a 4.3% increase in the probability of LBW among the sample. Lastly, a four-policy increase in a state’s restrictiveness index among those attending some college increased the probability of LBW by 0.15 percentage points (95% CI: 0.04, 0.27; *p* < 0.01) compared to college graduates, a 1.8% increase in the probability of LBW among the sample.

**Fig. 2:**
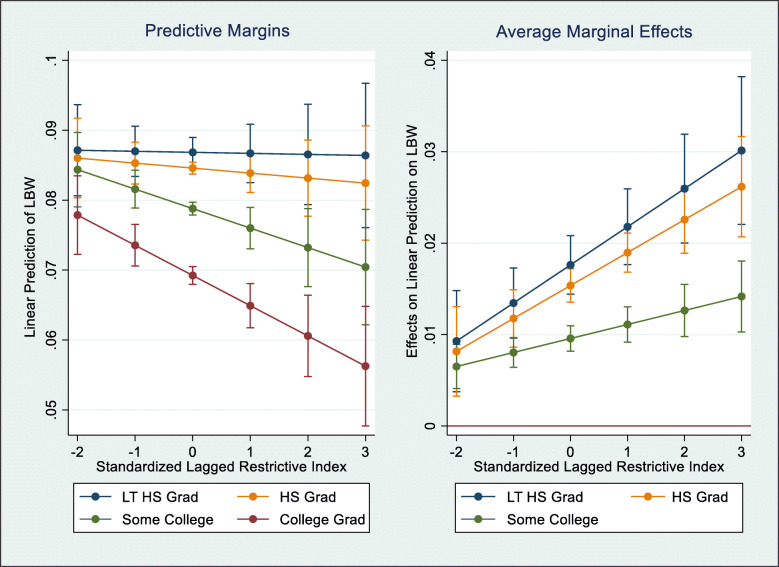
Predictive Margins and Average Marginal Effects of Educational Inequities in Relationship between Restrictiveness Index and Low Birthweight. Note: Results are predictive margins and average marginal effects from multivariate linear probability models estimating the moderating effect of education level on the relationship between the standardized lagged restrictiveness index and the probability of low birthweight among all 50 states and Washington, D.C. Final sample size included people not missing any data on education level, restrictiveness index, low birthweight, and covariates. All models adjust for individual-level sociodemographic characteristics, state-level sociodemographic, economic, and political characteristics, and state and year fixed effects. Standard errors clustered at the state level

These effects are presented as predictive margins in Table 3 and the left panel of Fig. 2, where we see the predicted values of LBW for each education level, which decline with additional years of education. In the least restrictive states (− 1 SD), the predicted LBW values were 8.7% for those with less than a high school degree, 8.5% for high school graduates, 8.2% for those with some college, and 7.4% for college graduates. These inequities worsened as states grew increasingly restrictive; while predicted LBW values remained approximately stable for those with less than a high school degree and high school graduates, they declined by nearly one percentage point for those with some college and by 1.3 percentage points for college graduates. Indeed, in the most restrictive states (+ 2 SD), the predicted LBW values were 8.7% for those with less than a high school degree, 8.3% for high school graduates, 7.3% for those with some college, and 6.1% for college graduates. The right panel of Fig. 2 presents the average marginal effects of different education levels (versus college graduates) on the relationship between the restrictiveness index and the predicted probability of LBW. As with the predictive margins, we see how the effect of having less than a high school degree, having a high school degree, or having attended some college (versus having graduated college) on the predicted probability of LBW increased as states grew increasingly restrictive. The inequities between college graduates and all other educational levels were inversely proportional to years of education, with the most severe inequity existing between college graduates and those with less than a high school degree.

### Moderation by race/ethnicity and education

Although not presented here, we further investigated the potential for moderation of the relationship between restrictive abortion policies and adverse birth outcomes by race/ethnicity and education via a three-way interaction (see Supplemental Tables 4, 5, 6 and Supplemental Figs. 4, 5, 6, and 7). Briefly, the three-way interaction revealed that increasingly restrictive abortion landscape predicted the likelihood of PTB and LBW to a greater extent for Black birthing people, compared to people of other racial/ethnic identities, across all education levels.

## Discussion

State policymakers have increasingly used policy as a lever to regulate access to abortion services across the United States [2, 6, 42, 60]. Recent evidence highlights the rise of PTB [104, 105] and LBW [106] rates in the United States, specifically among Black and lower SES populations. Restrictive abortion policies may be one mechanism contributing to these adverse birth outcomes. Given the vast racial/ethnic, socioeconomic, and intersecting racial/ethnic *and* socioeconomic inequities in U.S. birth outcomes [70–86, 88–92, 107, 108] and abortion rates [61–64], along with the knowledge that structural factors directly influence health and health inequities [35, 55–58], we sought to examine how the relationship between a state’s environment towards abortion access and two key adverse birth outcomes were moderated by race/ethnicity and education level.

Analyses revealed that the relationship between the restrictiveness index and the probability of adverse birth outcomes varied by racial/ethnic identity and education level. For Black individuals, increased exposure to restrictive abortion policies was associated with a 3% higher probability of PTB compared to non-Black individuals. For those with less than a college degree, increased exposure to restrictive abortion policies was associated with a 2 to 5% higher probability of LBW compared to college graduates. In both instances, the inequities between Black and non-Black individuals and those with fewer years of education and college graduates worsened as state environments became increasingly restrictive. With these findings, it is critical to acknowledge in our interpretations that an individual’s sociodemographic characteristics – in this case, identifying as Black or having fewer years of education – do not lead to poor health outcomes; the structural and systematic oppression and devaluation of Black and lower SES people lead to poor health outcomes [109].

Although these associations are small in magnitude, these findings have important implications for the health of infants born to Black birthing people and those with fewer years of education, particularly when examining them at the population level. PTB and LBW are two primary factors in infant mortality [110], accounting for 17% of infant deaths in 2017 [111]. Additionally, infants born preterm or LBW may be more likely to experience negative health and social outcomes as they age [105, 106, 110, 112], including respiratory, gastrointestinal, and cardiovascular disorders [110], decreased language skills and increased language delays [113–117], and diminished educational attainment [118]. Taken together, the weight of these consequences for infants born to Black individuals and those with fewer years of education are immense, particularly given the structural barriers to health, economic security, educational attainment, and access to care that systematically marginalized individuals are disproportionately forced to navigate. Furthermore, our findings suggest that restrictive abortion policies may exacerbate substantial and enduring racial and socioeconomic inequities in infant morbidity in the U.S.

Although no existing studies have explicitly examined the moderating effects of race/ethnicity and SES on the relationship between restrictive abortion policies and adverse birth outcomes, these findings support existing literature examining how restrictive abortion and reproductive rights policies influence racial and socioeconomic groups differentially. In a pre-post analysis of a restrictive medication abortion policy in Ohio, Upadhyay and colleagues found that, following policy implementation, fewer patients were Black and had lower levels of education [67]. Upon examining fluctuations in Medicaid funding for abortion services in North Carolina, Cook et al. noted that the absence of Medicaid abortion funding was associated with 10% fewer abortions for Black individuals and 1% fewer abortions for White individuals [68]. A national analysis by Coles et al. revealed that Black teenagers living in states with Medicaid funding restrictions were four times more likely to experience an unintended birth [69]. Lastly, Sudhinaraset and colleagues found that LBW risk was 8% lower among Black women living in states with the least restrictive reproductive rights policies, compared to counterparts living in the most restrictive states [53].

These findings encourage further research on sociodemographic inequities in the effects of restrictive abortion policies and other structural determinants of health. For instance, a substantial literature base examines the implications of restrictive abortion policies, yet the vast majority of these studies have not examined sociodemographic inequities in their analyses via estimating moderation effects [119]. The implications of these inequities should also be explored qualitatively, centering the experiences of Black and lower SES individuals and communities who experience adverse birth outcomes due to restrictive abortion policies and other structural determinants of health. Additionally, this work highlights the opportunity to study state policies seeking to improve maternal and infant health and health equity, an area that has received relatively less attention in recent years (compared to research on restrictive policy environments).

### Policy implications

As structural determinants of health, restrictive abortion policies are “upstream determinants … that shape who has access to health-promoting resources and opportunities” [35]. Our findings – that restrictive abortion policies disproportionately and negatively affect the health of Black individuals and individuals with fewer years of education – highlight the inherent devaluation of Black and lower educated bodies centered at the root of restrictive abortion policies [33, 120]. In order to combat the perpetuation of these white supremacist values, policymakers at the local, state, and federal levels must prioritize enacting structural interventions to center and protect the health of Black birthing people and those with fewer years of education. Such legislation should focus on dismantling, rebuilding, or redesigning structural systems in order to redistribute resources and opportunities more equitably. At the state level, legislative priorities could include efforts to expand postpartum Medicaid coverage [121, 122], Medicaid coverage of doula services [123], and shifting to community-informed models of perinatal and reproductive health care [124, 125]. At the federal level, an excellent example is the *Black Maternal Health Momnibus Act of 2021*, an act introduced by Congresswomen Alma Adams and Lauren Underwood, Senator Cory Booker, and members of the Black Maternal Health Caucus to address the maternal health crisis in America. Among its many provisions, the *Momnibus Act* seeks to “make critical investments in social determinants of health that influence maternal health outcomes, like housing, transportation, and nutrition,” “provide funding to community-based organizations … working to improve maternal health outcomes and promote equity,” and “grow and diversify the perinatal workforce to ensure that every mom in America receives culturally congruent maternity care and support” [126].

### Limitations

Although this methodologically rigorous analysis fills critical conceptual gaps in the abortion policy evidence base, it is not without limitations. First, because the restrictiveness index represents a sum of the number of restrictive abortion policies present in a given state during a given year, this measure does not isolate effects of specific restrictive abortion policies, nor does it allow for identification or exploration of the specific causal pathways through which restrictive abortion policies may influence adverse birth outcomes. Furthermore, the composite restrictiveness index treats all policies included as equivalent and does not reflect varied levels of restrictiveness associated with different restrictive abortion policies. However, as our goal was to assess inequities in the relationship between the restrictiveness of an environment toward abortion access on adverse birth outcomes, we that determined the restrictiveness index represented an adequate methodological solution. Second, because educational attainment does not capture information about the quality of an education [127], the neighborhood one lives in, or the financial resources of one’s family, community, and state [128–130], it is not a universally comparable indicator of SES. However, data on income – another commonly used indicator of SES [65, 74, 131] – were inconsistently available in the NCHS data files. Lastly, although we included a robust set of individual- and time-varying state-level covariates in these models, this study may be vulnerable to unmeasured time-varying confounding as a limitation of fixed-effects modeling [132].

## Conclusions

Due to the inequitable nature of the economic, political, social, and healthcare systems in the United States, restrictive abortion policies disproportionately affect more vulnerable groups, potentially causing or worsening health inequities. This study examines the differential associations of restrictive abortion policies on adverse birth outcomes. Our findings suggest that Black individuals and those with fewer years of education disproportionately experience negative birth outcomes as exposure to restrictive abortion policies increased, and that these inequities worsen as states grow increasingly restrictive. These findings suggest that restrictive abortion policies may contribute to increases in PTB [104, 105] and LBW [106] rates across the U.S. while simultaneously compounding racial/ethnic and socioeconomic inequities in infant health. As such, this study has important implications for policymakers, who should prioritize enacting policies addressing structural inequities in health and healthcare in order to combat the devaluation of Black and lower educated bodies in the legislative sphere.

## Supplementary Information

**Additional file 1: Supplemental Table 1**. Linear Probability Models Examining Moderating Effect of Race/Ethnicity (Categorical) on Relationship between Restrictiveness Index and Adverse Birth Outcomes: Linked Birth Infant Death Files, 2005-2015. **Supplemental Table 2**. Linear Probability Models Examining Moderating Effect of Race/Ethnicity (Dichotomous) on Relationship between Restrictiveness Index and Adverse Birth Outcomes: Linked Birth Infant Death Files, 2005-2015. **Supplemental Table 3**. Linear Probability Models Examining Moderating Effect of Education Level on Relationship between Restrictiveness Index and Adverse Birth Outcomes: Linked Birth Infant Death Files, 2005-2015. **Supplemental Table 4**. Linear Probability Models Examining Moderating Effect of Race/Ethnicity (Categorical) and Education Level on Relationship between Restrictiveness Index and Adverse Birth Outcomes: Linked Birth Infant Death Files, 2005-2015. **Supplemental Table 5**. Linear Probability Models Examining Moderating Effect of Race/Ethnicity (Dichotomous) and Education Level on Relationship between Restrictiveness Index and Adverse Birth Outcomes: Linked Birth Infant Death Files, 2005-2015. **Supplemental Table 6**. Predictive Margins of Preterm Birth and Low Birthweight from Linear Probability Models Examining Moderating Effects of Race/Ethnicity and Education Level on Relationship between Restrictiveness Index and Adverse Birth Outcomes.

**Additional file 2: Supplemental Figure 1**. Predictive Margins and Average Marginal Effects of Racial/Ethnic (Categorical) Inequities in Relationship between Restrictiveness Index and Adverse Birth Outcomes. **Supplemental Figure 2**. Predictive Margins and Average Marginal Effects of Racial/Ethnic (Dichotomous) Inequities in Relationship between Restrictiveness Index and Low Birthweight. **Supplemental Figure 3**. Predictive Margins and Average Marginal Effects of Educational Inequities in Relationship between Restrictiveness Index and Preterm Birth. **Supplemental Figure 4**. Predictive Margins and Average Marginal Effects of (Categorical) Racial/Ethnic-Educational Inequities in Relationship between Restrictiveness Index and Preterm Birth. **Supplemental Figure 5**. Predictive Margins and Average Marginal Effects of (Categorical) Racial/Ethnic-Educational Inequities in Relationship between Restrictiveness Index and Low Birthweight. **Supplemental Figure 6**. Predictive Margins and Average Marginal Effects of (Dichotomous) Racial/Ethnic-Educational Inequities in Relationship between Restrictiveness Index and Preterm Birth. **Supplemental Figure 7**. Predictive Margins and Average Marginal Effects of (Dichotomous) Racial/Ethnic-Educational Inequities in Relationship between Restrictiveness Index and Low Birthweight.

## Data Availability

The data that support the findings of this study are available from the National Center for Health Statistics but restrictions apply to the availability of these micro-data, which were used under license for the current study, and so are not publicly available. Researchers interested in applying for access to restricted-use micro-data files should contact the National Center for Health Statistics.

## Abbreviations

AAPI: Asian American / Pacific Islander
AIAN: American Indian / Alaska Native
LBW: Low birthweight
NCHS: National Center for Health Statistics
PTB: Preterm birth
SD: Standard deviation
SES: Socioeconomic status

## Footnotes

1 Interaction plots for models with non-significant interaction terms are presented in Supplemental Figures 1, 2, and 3.

2 Although the regression coefficient for RI*Black (non-Hispanic) was statistically significant at *p* < 0.05, the interaction term as a whole was not significant.
