# Supplementary material for: Racial/ethnic and educational inequities in restrictive abortion policy variation and adverse birth outcomes in the United States
Source: BMC Health Serv Res. 2021 Oct 22;21:1139. doi: 10.1186/s12913-021-07165-x (PMC8532280; doi:10.1186/s12913-021-07165-x)
Supplement: Supplementary file 1 — Additional file 1: Supplemental Table 1. Linear Probability Models Examining Moderating Effect of Race/Ethnicity (Categorical) on Relationship between Restrictiveness Index and Adverse Birth Outcomes: Linked Birth Infant Death Files, 2005-2015. Supplemental Table 2. Linear Probability Models Examining Moderating Effect of Race/Ethnicity (Dichotomous) on Relationship between Restrictiveness Index and Adverse Birth Outcomes: Linked Birth Infant Death Files, 2005-2015. Supplemental Table 3. Linear Probability Models Examining Moderating Effect of Education Level on Relationship between Restrictiveness Index and Adverse Birth Outcomes: Linked Birth Infant Death Files, 2005-2015. Supplemental Table 4. Linear Probability Models Examining Moderating Effect of Race/Ethnicity (Categorical) and Education Level on Relationship between Restrictiveness Index and Adverse Birth Outcomes: Linked Birth Infant Death Files, 2005-2015. Supplemental Table 5. Linear Probability Models Examining Moderating Effect of Race/Ethnicity (Dichotomous) and Education Level on Relationship between Restrictiveness Index and Adverse Birth Outcomes: Linked Birth Infant Death Files, 2005-2015. Supplemental Table 6. Predictive Margins of Preterm Birth and Low Birthweight from Linear Probability Models Examining Moderating Effects of Race/Ethnicity and Education Level on Relationship between Restrictiveness Index and Adverse Birth Outcomes. [file 12913_2021_7165_MOESM1_ESM.docx]

Supplemental Table 1. Linear Probability Models Examining Moderating Effect of Race/Ethnicity (Categorical) on Relationship between Restrictiveness Index and Adverse Birth Outcomes: Linked Birth Infant Death Files, 2005-2015

|  | (1) | (2) |
| --- | --- | --- |
|  | *Preterm Birth* | *Low Birthweight* |
| *Interaction Term* |  |  |
| **Standardized Lagged Restrictiveness Index (RI)*Race/ethnicity (categorical)** | | |
| RI*AIAN (Non-Hispanic) | -0.00243 [-0.00920, 0.00433] | 0.0000550 [-0.00460, 0.00471] |
| RI*AAPI (Non-Hispanic) | -0.00173 [-0.00399, 0.000526] | 0.000101 [-0.00137, 0.00157] |
| RI*Black (Non-Hispanic) | 0.00247^*^ [0.000145, 0.00480] | 0.00225 [-0.000804, 0.00531] |
| RI*Hispanic/Latinx | -0.00140 [-0.00406, 0.00126] | -0.00171 [-0.00371, 0.000281] |
| RI*White (Non-Hispanic) | Ref. | Ref. |
| *Restrictiveness Index and Race/ethnicity* | | |
| **Standardized Lagged Restrictiveness Index** | -0.000731 [-0.00332, 0.00185] | -0.00222 [-0.00520, 0.000753] |
| **Race/ethnicity (categorical)** |  |  |
| AIAN (Non-Hispanic) | 0.000914 [-0.00575, 0.00758] | -0.0127^**^ [-0.0206, -0.00477] |
| AAPI (Non-Hispanic) | 0.00515^***^ [0.00269, 0.00761] | 0.0193^***^ [0.0176, 0.0210] |
| Black (Non-Hispanic) | 0.0351^***^ [0.0315, 0.0387] | 0.0413^***^ [0.0372, 0.0454] |
| Hispanic/Latinx | 0.00293 [-0.00162, 0.00747] | -0.00492^*^ [-0.00882, -0.00103] |
| White (Non-Hispanic) | Ref. | Ref. |
| *Individual-level Covariates* |  |  |
| **Education** |  |  |
| Less than HS grad | 0.0184^***^ [0.0154, 0.0214] | 0.0175^***^ [0.0136, 0.0214] |
| HS grad | 0.0164^***^ [0.0141, 0.0188] | 0.0154^***^ [0.0126, 0.0181] |
| Some college | 0.0125^***^ [0.0104, 0.0146] | 0.00950^***^ [0.00790, 0.0111] |
| College grad or more | Ref. | Ref. |
| **Age** |  |  |
| 19 years and under | 0.00932^***^ [0.00775, 0.0109] | 0.00425^***^ [0.00258, 0.00592] |
| 20-29 years | Ref. | Ref. |
| 30-39 years | 0.0128^***^ [0.0119, 0.0138] | 0.00719^***^ [0.00627, 0.00811] |
| 40+ years | 0.0426^***^ [0.0393, 0.0459] | 0.0293^***^ [0.0265, 0.0320] |
| **Married** | -0.0151^***^ [-0.0172, -0.0130] | -0.0138^***^ [-0.0160, -0.0117] |
| **Previous Live Births** |  |  |
| 1 or 2 | Ref. | Ref. |
| 3 or 4 | 0.00479^***^ [0.00317, 0.00640] | -0.0119^***^ [-0.0129, -0.0110] |
| 5 or more | 0.0133^***^ [0.00786, 0.0187] | -0.0103^***^ [-0.0130, -0.00756] |
| **Prenatal Care Visits** | -0.00988^***^ [-0.0107, -0.00902] | -0.00713^***^ [-0.00779, -0.00647] |
| **Diabetes** | 0.0398^***^ [0.0371, 0.0426] | 0.00794^***^ [0.00514, 0.0107] |
| **Eclampsia** | 0.223^***^ [0.206, 0.240] | 0.216^***^ [0.197, 0.236] |
| **Chronic Hypertension** | 0.120^***^ [0.114, 0.125] | 0.107^***^ [0.102, 0.113] |
| **Pregnancy-associated Hypertension** | 0.123^***^ [0.115, 0.132] | 0.113^***^ [0.105, 0.122] |
| **Infant Sex (Female)** | -0.00994^***^ [-0.0109, -0.00896] | 0.0126^***^ [0.0109, 0.0143] |
| **Singleton Birth** | -0.492^***^ [-0.501, -0.482] | -0.515^***^ [-0.524, -0.506] |
| *State-level Covariates* |  |  |
| **High School**  **Graduates (%)** | -0.00294^**^ [-0.00495, -0.000931] | -0.00227^**^ [-0.00365, -0.000886] |
| **Marriage (%)** | -0.000940 [-0.00288, 0.000997] | -0.000586 [-0.00176, 0.000590] |
| **Non-white (%)** | -0.000146 [-0.000964, 0.000673] | -0.000188 [-0.000765, 0.000389] |
| **Poverty (%)** | -0.00164 [-0.00396, 0.000687] | 0.0000707 [-0.00123, 0.00138] |
| **Unemployment (%)** | -0.000775 [-0.00191, 0.000355] | -0.000912 [-0.00187, 0.0000502] |
| **Uninsured (%)** | 0.000756 [-0.000176, 0.00169] | 0.000306 [-0.000318, 0.000930] |
| **Avg. Mo. TANF**  **Payment ($)** | 0.00000832 [-0.0000280, 0.0000447] | 0.000000585 [-0.0000220, 0.0000231] |
| **Partisan Composition** |  |  |
| Democrat | 0.00330 [-0.00110, 0.00770] | 0.000899 [-0.00204, 0.00383] |
| Republican | Ref. | Ref. |
| Split/Unicameral | 0.000666 [-0.00258, 0.00391] | -0.00132 [-0.00338, 0.000733] |
| **Medicaid Expansion** | -0.000716 [-0.00337, 0.00194] | -0.000494 [-0.00226, 0.00128] |
| **Medicaid Family Planning Expansion** | 0.000465 [-0.00254, 0.00347] | -0.000211 [-0.00173, 0.00131] |
| *Wald Test of Interaction Term* | Χ^2^ = 1.69,  p = 0.1681 | Χ^2^ = 2.01,  p = 0.1068 |
| Conditional Mean | 0.1199 | 0.0813 |
| Sample Size | 2,058,512 | 2,061,512 |

Note: Results are marginal effects and 95% confidence intervals (CIs) from multivariate linear probability models estimating the moderating effect of race/ethnicity (categorical) on the relationship between the standardized lagged restrictiveness index and the probabilities of preterm birth and low birthweight among all 50 states and Washington, D.C. Final sample sizes included people who were not missing any data on race/ethnicity, restrictiveness index, outcomes, and covariates. All models adjust for individual-level sociodemographic characteristics, state-level sociodemographic, economic, and political characteristics, and state and year fixed effects. Standard errors clustered at the state level. Wald test of interaction term tests if the interaction term as a whole is statistically significant. Mean probability of outcomes listed above sample size (“conditional mean”). *p*-values significant at ^*^ *p* <.05, ^**^ *p* <.01, ^***^ *p* <.001. AIAN = American Indian / Alaska Native; AAPI = Asian American / Pacific Islander; HS = high school; GED = general educational development diploma; TANF = Temporary Assistance for Needy Families

Supplemental Table 2. Linear Probability Models Examining Moderating Effect of Race/Ethnicity (Dichotomous) on Relationship between Restrictiveness Index and Adverse Birth Outcomes: Linked Birth Infant Death Files, 2005-2015

|  | (1) | (2) |
| --- | --- | --- |
|  | *Preterm Birth* | *Low Birthweight* |
| *Interaction Term* |  |  |
| **Standardized Lagged Restrictiveness Index (RI)*Race/ethnicity (dichotomous)** | | |
| RI*Black (Non-Hispanic) | 0.00334^*^ [0.000809, 0.00587] | 0.00292 [-0.000134, 0.00597] |
| RI*Non-Black | Ref. | Ref. |
| *Restrictiveness Index and Race/ethnicity* | | |
| **Standardized Lagged Restrictiveness Index** | -0.00129 [-0.00393, 0.00135] | -0.00272^*^ [-0.00541, -0.0000202] |
| **Race/ethnicity (dichotomous)** |  |  |
| Black (Non-Hispanic) | 0.0338^***^ [0.0301, 0.0375] | 0.0420^***^ [0.0381, 0.0460] |
| Non-Black | Ref. | Ref. |
| *Individual-level Covariates* |  |  |
| **Education** |  |  |
| Less than HS grad | 0.0194^***^ [0.0171, 0.0217] | 0.0147^***^ [0.0115, 0.0179] |
| HS grad | 0.0168^***^ [0.0148, 0.0187] | 0.0136^***^ [0.0110, 0.0162] |
| Some college | 0.0126^***^ [0.0107, 0.0145] | 0.00813^***^ [0.00660, 0.00966] |
| College grad or more | Ref. | Ref. |
| **Age** |  |  |
| 19 years and under | 0.00915^***^ [0.00756, 0.0107] | 0.00426^***^ [0.00273, 0.00579] |
| 20-29 years | Ref. | Ref. |
| 30-39 years | 0.0129^***^ [0.0120, 0.0139] | 0.00757^***^ [0.00665, 0.00848] |
| 40+ years | 0.0426^***^ [0.0393, 0.0459] | 0.0298^***^ [0.0269, 0.0326] |
| **Married** | -0.0152^***^ [-0.0173, -0.0131] | -0.0128^***^ [-0.0148, -0.0108] |
| **Previous Live Births** |  |  |
| 1 or 2 | Ref. | Ref. |
| 3 or 4 | 0.00476^***^ [0.00314, 0.00639] | -0.0127^***^ [-0.0136, -0.0117] |
| 5 or more | 0.0131^***^ [0.00765, 0.0186] | -0.0112^***^ [-0.0138, -0.00849] |
| **Prenatal Care Visits** | -0.00990^***^ [-0.0108, -0.00904] | -0.00715^***^ [-0.00780, -0.00651] |
| **Diabetes** | 0.0401^***^ [0.0375, 0.0428] | 0.00859^***^ [0.00557, 0.0116] |
| **Eclampsia** | 0.223^***^ [0.206, 0.240] | 0.216^***^ [0.196, 0.235] |
| **Chronic Hypertension** | 0.119^***^ [0.114, 0.125] | 0.107^***^ [0.102, 0.112] |
| **Pregnancy-associated Hypertension** | 0.123^***^ [0.115, 0.132] | 0.113^***^ [0.104, 0.122] |
| **Infant Sex (Female)** | -0.00994^***^ [-0.0109, -0.00896] | 0.0126^***^ [0.0109, 0.0143] |
| **Singleton Birth** | -0.491^***^ [-0.501, -0.482] | -0.515^***^ [-0.524, -0.506] |
| *State-level Covariates* |  |  |
| **High School**  **Graduates (%)** | -0.00297^**^ [-0.00496, -0.000966] | -0.00229^**^ [-0.00368, -0.000907] |
| **Marriage (%)** | -0.000939 [-0.00287, 0.000995] | -0.000560 [-0.00173, 0.000609] |
| **Non-white (%)** | -0.000129 [-0.000942, 0.000684] | -0.000172 [-0.000750, 0.000406] |
| **Poverty (%)** | -0.00164 [-0.00395, 0.000668] | 0.0000842 [-0.00120, 0.00137] |
| **Unemployment (%)** | -0.000795 [-0.00193, 0.000335] | -0.000925 [-0.00188, 0.0000346] |
| **Uninsured (%)** | 0.000771 [-0.000153, 0.00170] | 0.000310 [-0.000305, 0.000926] |
| **Avg. Mo. TANF**  **Payment ($)** | 0.00000776 [-0.0000287, 0.0000442] | 0.000000210 [-0.0000223, 0.0000228] |
| **Partisan Composition** |  |  |
| Democrat | 0.00322 [-0.00119, 0.00763] | 0.000831 [-0.00209, 0.00375] |
| Republican | Ref. | Ref. |
| Split/Unicameral | 0.000602 [-0.00267, 0.00387] | -0.00140 [-0.00341, 0.000621] |
| **Medicaid Expansion** | -0.000708 [-0.00337, 0.00196] | -0.000430 [-0.00218, 0.00132] |
| **Medicaid Family Planning Expansion** | 0.000476 [-0.00254, 0.00349] | -0.000229 [-0.00175, 0.00129] |
| *Wald Test of Interaction Term* | Χ^2^ = 7.02,  p = 0.0107 | Χ^2^ = 3.69,  p = 0.0606 |
| Conditional Mean | 0.1199 | 0.0813 |
| Sample Size | 2,058,512 | 2,061,512 |

Note: Results are marginal effects and 95% confidence intervals (CIs) from multivariate linear probability models estimating the moderating effect of race/ethnicity (dichotomous) on the relationship between the standardized lagged restrictiveness index and the probabilities of preterm birth and low birthweight among all 50 states and Washington, D.C. Final sample sizes included people who were not missing any data on race/ethnicity, restrictiveness index, outcomes, and covariates. All models adjust for individual-level sociodemographic characteristics, state-level sociodemographic, economic, and political characteristics, and state and year fixed effects. Standard errors clustered at the state level. Wald test of interaction term tests if the interaction term as a whole is statistically significant. Mean probability of outcomes listed above sample size (“conditional mean”). *p*-values significant at ^*^ *p* <.05, ^**^ *p* <.01, ^***^ *p* <.001. HS = high school; GED = general educational development diploma; TANF = Temporary Assistance for Needy Families

Supplemental Table 3. Linear Probability Models Examining Moderating Effect of Education Level on Relationship between Restrictiveness Index and Adverse Birth Outcomes: Linked Birth Infant Death Files, 2005-2015

|  | (1) | (2) |
| --- | --- | --- |
|  | *Preterm Birth* | *Low Birthweight* |
| *Interaction Term* |  |  |
| **Standardized Lagged Restrictiveness Index (RI)*Education** | | |
| RI*Less than HS | 0.00190 [-0.000200, 0.00399] | 0.00417^***^ [0.00178, 0.00656] |
| RI*HS grad | 0.00241^*^ [0.000446, 0.00436] | 0.00360^***^ [0.00164, 0.00557] |
| RI*Some college | 0.00186^*^ [0.000327, 0.00340] | 0.00153^**^ [0.000407, 0.00266] |
| RI*College grad | Ref. | Ref. |
| *Restrictiveness Index and Education* | | |
| **Standardized Lagged Restrictiveness Index** | -0.00235 [-0.00491, 0.000220] | -0.00432^**^ [-0.00711, -0.00153] |
| **Education** |  |  |
| Less than HS grad | 0.0185^***^ [0.0153, 0.0216] | 0.0176^***^ [0.0144, 0.0208] |
| HS grad | 0.0165^***^ [0.0144, 0.0185] | 0.0154^***^ [0.0135, 0.0172] |
| Some college | 0.0126^***^ [0.0106, 0.0145] | 0.00956^***^ [0.00817, 0.0110] |
| College grad or more | Ref. | Ref. |
| *Individual Characteristics* |  |  |
| **Race/ethnicity** |  |  |
| AIAN (Non-Hispanic) | 0.000712 [-0.00645, 0.00787] | -0.0127^**^ [-0.0206, -0.00477] |
| AAPI (Non-Hispanic) | 0.00561^***^ [0.00307, 0.00815] | 0.0187^***^ [0.0164, 0.0209] |
| Black (Non-Hispanic) | 0.0356^***^ [0.0320, 0.0392] | 0.0417^***^ [0.0381, 0.0453] |
| Hispanic/Latinx | 0.00354 [-0.000850, 0.00792] | -0.00393 [-0.00842, 0.000562] |
| White (Non-Hispanic) | Ref. | Ref. |
| **Age** |  |  |
| 19 years and under | 0.00929^***^ [0.00772, 0.0109] | 0.00412^***^ [0.00247, 0.00578] |
| 20-29 years | Ref. | Ref. |
| 30-39 years | 0.0128^***^ [0.0119, 0.0137] | 0.00711^***^ [0.00618, 0.00804] |
| 40+ years | 0.0424^***^ [0.0391, 0.0457] | 0.0290^***^ [0.0263, 0.0318] |
| **Married** | -0.0151^***^ [-0.0173, -0.0129] | -0.0138^***^ [-0.0160, -0.0116] |
| **Previous Live Births** |  |  |
| 1 or 2 | Ref. | Ref. |
| 3 or 4 | 0.00483^***^ [0.00322, 0.00645] | -0.0118^***^ [-0.0128, -0.0108] |
| 5 or more | 0.0134^***^ [0.00792, 0.0188] | -0.0102^***^ [-0.0130, -0.00748] |
| **Prenatal Care Visits** | -0.00988^***^ [-0.0107, -0.00902] | -0.00712^***^ [-0.00779, -0.00645] |
| **Diabetes** | 0.0398^***^ [0.0370, 0.0425] | 0.00792^***^ [0.00514, 0.0107] |
| **Eclampsia** | 0.223^***^ [0.206, 0.240] | 0.216^***^ [0.197, 0.236] |
| **Chronic Hypertension** | 0.120^***^ [0.114, 0.125] | 0.108^***^ [0.102, 0.113] |
| **Pregnancy-associated Hypertension** | 0.123^***^ [0.115, 0.132] | 0.114^***^ [0.105, 0.122] |
| **Infant Sex (Female)** | -0.00994^***^ [-0.0109, -0.00896] | 0.0126^***^ [0.0109, 0.0143] |
| **Singleton Birth** | -0.492^***^ [-0.501, -0.482] | -0.515^***^ [-0.524, -0.506] |
| *State Characteristics* |  |  |
| **High School**  **Graduates (%)** | -0.00287^**^ [-0.00487, -0.000873] | -0.00217^**^ [-0.00354, -0.000805] |
| **Marriage (%)** | -0.000965 [-0.00289, 0.000963] | -0.000629 [-0.00179, 0.000535] |
| **Non-white (%)** | -0.000121 [-0.000928, 0.000685] | -0.000139 [-0.000708, 0.000430] |
| **Poverty (%)** | -0.00162 [-0.00394, 0.000705] | 0.0000566 [-0.00122, 0.00134] |
| **Unemployment (%)** | -0.000796 [-0.00193, 0.000336] | -0.000950^*^ [-0.00189, -0.0000128] |
| **Uninsured (%)** | 0.000792 [-0.000134, 0.00172] | 0.000378 [-0.000231, 0.000986] |
| **Avg. Mo. TANF**  **Payment ($)** | 0.00000776 [-0.0000288, 0.0000443] | 0.000000477 [-0.0000219, 0.0000229] |
| **Partisan Composition** |  |  |
| Democrat | 0.00309 [-0.00128, 0.00747] | 0.000744 [-0.00213, 0.00361] |
| Republican | Ref. | Ref. |
| Split/Unicameral | 0.000541 [-0.00275, 0.00383] | -0.00143 [-0.00344, 0.000582] |
| **Medicaid Expansion** | -0.000834 [-0.00351, 0.00184] | -0.000682 [-0.00236, 0.000994] |
| **Medicaid Family Planning Expansion** | 0.000505 [-0.00251, 0.00352] | -0.000171 [-0.00167, 0.00132] |
| *Wald Test of Interaction Term* | Χ^2^ = 2.42,  p = 0.0766 | Χ^2^ = 5.17,  p = 0.0034 |
| Conditional Mean | 0.1199 | 0.0813 |
| Sample Size | 2,058,512 | 2,061,512 |

Note: Results are marginal effects and 95% confidence intervals (CIs) from multivariate linear probability models estimating the moderating effect of education level on the relationship between the standardized lagged restrictiveness index and the probabilities of preterm birth and low birthweight among all 50 states and Washington, D.C. Final sample sizes included people who were not missing any data on education level, restrictiveness index, outcomes, and covariates. All models adjust for individual-level sociodemographic characteristics, state-level sociodemographic, economic, and political characteristics, and state and year fixed effects. Standard errors clustered at the state level. Wald test of interaction term tests if the interaction term as a whole is statistically significant. Mean probability of outcomes above sample size (“conditional mean”). *p*-values significant at ^*^ *p* <.05, ^**^ *p* <.01, ^***^ *p* <.001. HS = high school; GED = general educational development diploma; AIAN = American Indian / Alaska Native; AAPI = Asian American / Pacific Islander; TANF = Temporary Assistance for Needy Families

Supplemental Table 4. Linear Probability Models Examining Moderating Effect of Race/Ethnicity (Categorical) and Education Level on Relationship between Restrictiveness Index and Adverse Birth Outcomes: Linked Birth Infant Death Files, 2005-2015

|  | (1) | (2) |
| --- | --- | --- |
|  | *Preterm Birth* | *Low Birthweight* |
| *Three-Way Interaction Term* |  |  |
| **Standardized Lagged Restrictiveness Index (RI)*Race/Ethnicity*Education** | | |
| RI*AIAN*Less than HS | 0.00671 [-0.0178,0.0312] | 0.00742 [-0.00299,0.0178] |
| RI*AIAN*HS grad | 0.000859 [-0.0155,0.0172] | 0.00399 [-0.00330,0.0113] |
| RI*AIAN*Some college | 0.00756 [-0.00591,0.0210] | 0.00658 [-0.00221,0.0154] |
| RI*AIAN*College grad | Ref. | Ref. |
| RI*AAPI*Less than HS | 0.00527 [-0.00247,0.0130] | -0.00307 [-0.0115,0.00537] |
| RI*AAPI*HS grad | 0.000169 [-0.00542,0.00575] | -0.00139 [-0.00560,0.00281] |
| RI*AAPI*Some college | 0.00428 [-0.000395,0.00895] | -0.000208 [-0.00294,0.00252] |
| RI*AAPI*College grad | Ref. | Ref. |
| RI*Black*Less than HS | 0.00836^**^ [0.00284,0.0139] | 0.00428 [-0.00211,0.0107] |
| RI*Black*HS grad | 0.0000360 [-0.00585,0.00592] | -0.00147 [-0.00713,0.00418] |
| RI*Black*Some college | 0.00142 [-0.00329,0.00614] | 0.00143 [-0.00216,0.00501] |
| RI*Black*College grad | Ref. | Ref. |
| RI*Hispanic/Latinx*Less than HS | 0.000180 [-0.00746,0.00782] | -0.00256 [-0.00803,0.00291] |
| RI*Hispanic/Latinx*HS grad | -0.00127 [-0.00798,0.00544] | -0.00228 [-0.00492,0.000371] |
| RI*Hispanic/Latinx*Some college | 0.00308 [-0.00262,0.00878] | -0.00000978 [-0.00255,0.00253] |
| RI*Hispanic/Latinx*College grad | Ref. | Ref. |
| RI*White*Less than HS | Ref. | Ref. |
| RI*White*HS grad |  |  |
| RI*White*Some college |  |  |
| RI*White*College grad |  |  |
| *Two-Way Interaction Terms* |  |  |
| **Standardized Lagged Restrictiveness Index (RI)*Race/ethnicity** |  |  |
| RI*AIAN (Non-Hispanic) | -0.00682 [-0.0168, 0.00314] | -0.00595 [-0.0130, 0.00105] |
| RI*AAPI (Non-Hispanic) | -0.00268^*^ [-0.00498, -0.000377] | 0.00132 [-0.00110, 0.00375] |
| RI*Black (Non-Hispanic) | 0.000190 [-0.00360, 0.00398] | 0.00108 [-0.00230, 0.00445] |
| RI*Hispanic/Latinx | -0.00162 [-0.00873, 0.00548] | -0.000625 [-0.00266, 0.00141] |
| RI*White (Non-Hispanic) | Ref. | Ref. |
| **Standardized Lagged Restrictiveness Index (RI)*Education** |  |  |
| RI*Less than HS | -0.000704 [-0.00407, 0.00266] | 0.00166 [-0.00292, 0.00625] |
| RI*HS grad | 0.00284^*^ [0.000438, 0.00525] | 0.00375^**^ [0.00103, 0.00646] |
| RI*Some college | 0.00131 [-0.000313, 0.00293] | 0.00150 [-0.0000869, 0.00309] |
| RI*College grad | Ref. | Ref. |
| **Race/ethnicity*Education** |  |  |
| AIAN*Less than HS | -0.000921 [-0.0211, 0.0192] | -0.0306^***^ [-0.0429, -0.0184] |
| AIAN*HS grad | -0.0112 [-0.0267, 0.00426] | -0.0272^***^ [-0.0372, -0.0172] |
| AIAN*Some college | -0.0103 [-0.0213, 0.000774] | -0.0154^**^ [-0.0263, -0.00453] |
| AIAN*College grad | Ref. | Ref. |
| AAPI*Less than HS | 0.00350 [-0.00698, 0.0140] | -0.0309^***^ [-0.0389, -0.0230] |
| AAPI*HS grad | 0.00442 [-0.00157, 0.0104] | -0.0150^***^ [-0.0204, -0.00964] |
| AAPI*Some college | 0.00725^**^ [0.00185, 0.0126] | -0.00464^**^ [-0.00782, -0.00147] |
| AAPI*College grad | Ref. | Ref. |
| Black*Less than HS | 0.00250 [-0.00310, 0.00810] | -0.00827^**^ [-0.0143, -0.00221] |
| Black*HS grad | 0.00241 [-0.00263, 0.00744] | 0.000317 [-0.00469, 0.00532] |
| Black*Some college | -0.000383 [-0.00556, 0.00479] | 0.00289 [-0.00207, 0.00785] |
| Black*College grad | Ref. | Ref. |
| Hispanic/Latinx*Less than HS | -0.0181^***^ [-0.0274, -0.00874] | -0.0353^***^ [-0.0408, -0.0298] |
| Hispanic/Latinx*HS grad | -0.00841^*^ [-0.0167, -0.000160] | -0.0157^***^ [-0.0185, -0.0130] |
| Hispanic/Latinx*Some college | -0.00200 [-0.00908, 0.00507] | -0.00369^**^ [-0.00619, -0.00119] |
| Hispanic/Latinx*College grad | Ref. | Ref. |
| White*Less than HS | Ref. | Ref. |
| White*HS grad |  |  |
| White*Some college |  |  |
| White*College grad |  |  |
| *Restrictiveness Index, Race/ethnicity, and Education* | | |
| **Standardized Lagged Restrictiveness Index** | -0.00174 [-0.00423, 0.000747] | -0.00367^*^ [-0.00666, -0.000680] |
| **Race/ethnicity** |  |  |
| AIAN (Non-Hispanic) | 0.00750 [-0.00310, 0.0181] | 0.00715 [-0.00282, 0.0171] |
| AAPI (Non-Hispanic) | 0.00275^*^ [0.000208, 0.00530] | 0.0256^***^ [0.0236, 0.0276] |
| Black (Non-Hispanic) | 0.0334^***^ [0.0292, 0.0377] | 0.0405^***^ [0.0370, 0.0440] |
| Hispanic/Latinx | 0.0116^*^ [0.00233, 0.0209] | 0.0102^***^ [0.00762, 0.0127] |
| White (Non-Hispanic) | Ref. | Ref. |
| **Education** |  |  |
| Less than HS grad | 0.0235^***^ [0.0191, 0.0279] | 0.0326^***^ [0.0288, 0.0364] |
| HS grad | 0.0168^***^ [0.0146, 0.0190] | 0.0186^***^ [0.0167, 0.0206] |
| Some college | 0.0123^***^ [0.0105, 0.0142] | 0.00942^***^ [0.00800, 0.0108] |
| College grad or more | Ref. | Ref. |
| *Individual Characteristics* |  |  |
| **Age** |  |  |
| 19 years and under | 0.00862^***^ [0.00706, 0.0102] | 0.00297^**^ [0.00123, 0.00472] |
| 20-29 years | Ref. | Ref. |
| 30-39 years | 0.0130^***^ [0.0121, 0.0140] | 0.00767^***^ [0.00673, 0.00861] |
| 40+ years | 0.0427^***^ [0.0394, 0.0460] | 0.0298^***^ [0.0270, 0.0326] |
| **Married** | -0.0150^***^ [-0.0172, -0.0128] | -0.0133^***^ [-0.0155, -0.0111] |
| **Previous Live Births** |  |  |
| 1 or 2 | Ref. | Ref. |
| 3 or 4 | 0.00489^***^ [0.00327, 0.00651] | -0.0118^***^ [-0.0128, -0.0108] |
| 5 or more | 0.0133^***^ [0.00781, 0.0187] | -0.0104^***^ [-0.0133, -0.00759] |
| **Prenatal Care Visits** | -0.00988^***^ [-0.0107, -0.00901] | -0.00712^***^ [-0.00779, -0.00645] |
| **Diabetes** | 0.0399^***^ [0.0372, 0.0427] | 0.00794^***^ [0.00518, 0.0107] |
| **Eclampsia** | 0.223^***^ [0.205, 0.240] | 0.216^***^ [0.197, 0.236] |
| **Chronic Hypertension** | 0.120^***^ [0.114, 0.125] | 0.107^***^ [0.102, 0.113] |
| **Pregnancy-associated Hypertension** | 0.123^***^ [0.115, 0.132] | 0.114^***^ [0.105, 0.122] |
| **Infant Sex (Female)** | -0.00994^***^ [-0.0109, -0.00895] | 0.0126^***^ [0.0110, 0.0143] |
| **Singleton Birth** | -0.492^***^ [-0.501, -0.482] | -0.515^***^ [-0.524, -0.506] |
| *State Characteristics* |  |  |
| **High School**  **Graduates (%)** | -0.00287^**^ [-0.00488, -0.000859] | -0.00214^**^ [-0.00352, -0.000754] |
| **Marriage (%)** | -0.000938 [-0.00287, 0.000994] | -0.000605 [-0.00177, 0.000565] |
| **Non-white (%)** | -0.0000874 [-0.000893, 0.000718] | -0.0000791 [-0.000639, 0.000481] |
| **Poverty (%)** | -0.00164 [-0.00397, 0.000695] | 0.0000377 [-0.00125, 0.00132] |
| **Unemployment (%)** | -0.000777 [-0.00191, 0.000360] | -0.000940 [-0.00189, 0.00000719] |
| **Uninsured (%)** | 0.000793 [-0.000138, 0.00172] | 0.000396 [-0.000224, 0.00101] |
| **Avg. Mo. TANF**  **Payment ($)** | 0.00000798 [-0.0000282, 0.0000441] | 0.00000139 [-0.0000209, 0.0000236] |
| **Partisan Composition** |  |  |
| Democrat | 0.00322 [-0.00121, 0.00764] | 0.000830 [-0.00210, 0.00376] |
| Republican | Ref. | Ref. |
| Split/Unicameral | 0.000614 [-0.00265, 0.00388] | -0.00137 [-0.00345, 0.000710] |
| **Medicaid Expansion** | -0.000786 [-0.00345, 0.00188] | -0.000623 [-0.00239, 0.00114] |
| **Medicaid Family Planning Expansion** | 0.000481 [-0.00254, 0.00350] | -0.000184 [-0.00169, 0.00132] |
| *Wald Test of Interaction Term* | Χ^2^ = 5.74,  p < 0.0001 | Χ^2^ = 2.03,  p = 0.0412 |
| Conditional Mean | 0.1199 | 0.0813 |
| Sample Size | 2,058,512 | 2,061,512 |

Note: Results are marginal effects and 95% confidence intervals (CIs) from multivariate linear probability models estimating the moderating effect of race/ethnicity (categorical) and education level on the relationship between the standardized lagged restrictiveness index and the probabilities of preterm birth and low birthweight among all 50 states and Washington, D.C. Final sample sizes included people who were not missing any data on race/ethnicity, education level, restrictiveness index, outcomes, and covariates. All models adjust for individual-level sociodemographic characteristics, state-level sociodemographic, economic, and political characteristics, and state and year fixed effects. Standard errors clustered at the state level. Wald test of interaction term tests if the interaction term as a whole is statistically significant. Mean probability of outcomes above sample size (“conditional mean”). *p*-values significant at ^*^ *p* <.05, ^**^ *p* <.01, ^***^ *p* <.001. AIAN = American Indian / Alaska Native; AAPI = Asian American / Pacific Islander; HS = high school; GED = general educational development diploma; TANF = Temporary Assistance for Needy Families

Supplemental Table 5. Linear Probability Models Examining Moderating Effect of Race/Ethnicity (Dichotomous) and Education Level on Relationship between Restrictiveness Index and Adverse Birth Outcomes: Linked Birth Infant Death Files, 2005-2015

|  | (1) | (2) |
| --- | --- | --- |
|  | *Preterm Birth* | *Low Birthweight* |
| *Three-Way Interaction Term* |  |  |
| **Standardized Lagged Restrictiveness Index (RI)*Race/Ethnicity*Education** | | |
| RI*Black*Less than HS | 0.00715^*^ [0.000968, 0.0133] | 0.000802 [-0.00591, 0.00752] |
| RI*Black*HS grad | 0.000801 [-0.00487, 0.00647] | -0.00252 [-0.00841, 0.00338] |
| RI*Black*Some college | 0.00108 [-0.00344, 0.00560] | 0.00102 [-0.00254, 0.00459] |
| RI*Black*College grad | Ref. | Ref. |
| RI*Non-Black*Less than HS | Ref. | Ref. |
| RI*Non-Black*HS grad |  |  |
| RI*Non-Black*Some college |  |  |
| RI*Non-Black*College grad |  |  |
| *Two-Way Interaction Terms* |  |  |
| **Standardized Lagged Restrictiveness Index (RI)*Race/ethnicity** |  |  |
| RI*Black (Non-Hispanic) | 0.00107 [-0.00263, 0.00477] | 0.00255 [-0.000818, 0.00592] |
| RI*White (Non-Hispanic) | Ref. | Ref. |
| **Standardized Lagged Restrictiveness Index (RI)*Education** |  |  |
| RI*Less than HS | 0.000497 [-0.00167, 0.00266] | 0.00502^**^ [0.00186, 0.00818] |
| RI*HS grad | 0.00208^*^ [0.000214, 0.00395] | 0.00471^**^ [0.00197, 0.00745] |
| RI*Some college | 0.00168^*^ [0.000245, 0.00311] | 0.00187^**^ [0.000484, 0.00325] |
| RI*College grad | Ref. | Ref. |
| **Race/ethnicity*Education** |  |  |
| Black*Less than HS | 0.00758^*^ [0.00129, 0.0139] | 0.0104^**^ [0.00337, 0.0173] |
| Black*HS grad | 0.00238 [-0.00255, 0.00732] | 0.00562^*^ [0.000385, 0.0109] |
| Black*Some college | -0.00123 [-0.00588, 0.00343] | 0.00442 [-0.000458, 0.00930] |
| Black*College grad | Ref. | Ref. |
| Non-Black*Less than HS | Ref. | Ref. |
| Non-Black*HS grad |  |  |
| Non-Black*Some college |  |  |
| Non-Black*College grad |  |  |
| *Restrictiveness Index, Race/ethnicity, and Education* | | |
| **Standardized Lagged Restrictiveness Index** | -0.00237 [-0.00490, 0.000170] | -0.00514^**^ [-0.00809, -0.00218] |
| **Race/ethnicity** |  |  |
| Black (Non-Hispanic) | 0.0318^***^ [0.0277, 0.0359] | 0.0366^***^ [0.0330, 0.0403] |
| Non-Black | Ref. | Ref. |
| **Education** |  |  |
| Less than HS grad | 0.0181^***^ [0.0154, 0.0208] | 0.0139^***^ [0.0110, 0.0168] |
| HS grad | 0.0166^***^ [0.0149, 0.0183] | 0.0134^***^ [0.0119, 0.0149] |
| Some college | 0.0130^***^ [0.0114, 0.0147] | 0.00800^***^ [0.00680, 0.00920] |
| College grad or more | Ref. | Ref. |
| *Individual Characteristics* |  |  |
| **Age** |  |  |
| 19 years and under | 0.00886^***^ [0.00728, 0.0104] | 0.00385^***^ [0.00230, 0.00540] |
| 20-29 years | Ref. | Ref. |
| 30-39 years | 0.0129^***^ [0.0119, 0.0139] | 0.00750^***^ [0.00659, 0.00841] |
| 40+ years | 0.0426^***^ [0.0393, 0.0458] | 0.0296^***^ [0.0268, 0.0324] |
| **Married** | -0.0153^***^ [-0.0174, -0.0132] | -0.0128^***^ [-0.0148, -0.0108] |
| **Previous Live Births** |  |  |
| 1 or 2 | Ref. | Ref. |
| 3 or 4 | 0.00484^***^ [0.00320, 0.00649] | -0.0125^***^ [-0.0135, -0.0115] |
| 5 or more | 0.0132^***^ [0.00766, 0.0187] | -0.0111^***^ [-0.0138, -0.00843] |
| **Prenatal Care Visits** | -0.00990^***^ [-0.0108, -0.00904] | -0.00714^***^ [-0.00780, -0.00649] |
| **Diabetes** | 0.0402^***^ [0.0375, 0.0429] | 0.00863^***^ [0.00565, 0.0116] |
| **Eclampsia** | 0.223^***^ [0.206, 0.240] | 0.216^***^ [0.196, 0.235] |
| **Chronic Hypertension** | 0.119^***^ [0.114, 0.125] | 0.107^***^ [0.102, 0.113] |
| **Pregnancy-associated Hypertension** | 0.123^***^ [0.115, 0.132] | 0.113^***^ [0.104, 0.122] |
| **Infant Sex (Female)** | -0.00994^***^ [-0.0109, -0.00896] | 0.0126^***^ [0.0109, 0.0143] |
| **Singleton Birth** | -0.491^***^ [-0.501, -0.482] | -0.515^***^ [-0.524, -0.506] |
| *State Characteristics* |  |  |
| **High School**  **Graduates (%)** | -0.00293^**^ [-0.00494, -0.000927] | -0.00222^**^ [-0.00361, -0.000828] |
| **Marriage (%)** | -0.000940 [-0.00288, 0.000997] | -0.000591 [-0.00176, 0.000581] |
| **Non-white (%)** | -0.000122 [-0.000934, 0.000690] | -0.000138 [-0.000715, 0.000440] |
| **Poverty (%)** | -0.00165 [-0.00397, 0.000674] | 0.0000421 [-0.00124, 0.00133] |
| **Unemployment (%)** | -0.000790 [-0.00193, 0.000348] | -0.000949 [-0.00190, 0.00000467] |
| **Uninsured (%)** | 0.000786 [-0.000147, 0.00172] | 0.000379 [-0.000242, 0.00100] |
| **Avg. Mo. TANF**  **Payment ($)** | 0.00000751 [-0.0000289, 0.0000440] | 0.000000693 [-0.0000217, 0.0000231] |
| **Partisan Composition** |  |  |
| Democrat | 0.00323 [-0.00121, 0.00767] | 0.000904 [-0.00204, 0.00384] |
| Republican | Ref. | Ref. |
| Split/Unicameral | 0.000605 [-0.00269, 0.00390] | -0.00136 [-0.00341, 0.000691] |
| **Medicaid Expansion** | -0.000777 [-0.00347, 0.00192] | -0.000631 [-0.00239, 0.00113] |
| **Medicaid Family Planning Expansion** | 0.000490 [-0.00253, 0.00351] | -0.000206 [-0.00172, 0.00131] |
| *Wald Test of Interaction Term* | Χ^2^ = 4.80,  p = 0.0051 | Χ^2^ = 2.87,  p = 0.0456 |
| Conditional Mean | 0.1199 | 0.0813 |
| Sample Size | 2,058,512 | 2,061,512 |

Note: Results are marginal effects and 95% confidence intervals (CIs) from multivariate linear probability models estimating the moderating effect of race/ethnicity (dichotomous) and education level on the relationship between the standardized lagged restrictiveness index and the probabilities of preterm birth and low birthweight among all 50 states and Washington, D.C. Final sample sizes included people who were not missing any data on race/ethnicity, education level, restrictiveness index, outcomes, and covariates. All models adjust for individual-level sociodemographic characteristics, state-level sociodemographic, economic, and political characteristics, and state and year fixed effects. Standard errors clustered at the state level. Wald test of interaction term tests if the interaction term as a whole is statistically significant. Mean probability of outcomes above sample size (“conditional mean”). *p*-values significant at ^*^ *p* <.05, ^**^ *p* <.01, ^***^ *p* <.001. AIAN = American Indian / Alaska Native; AAPI = Asian American / Pacific Islander; HS = high school; GED = general educational development diploma; TANF = Temporary Assistance for Needy Families

**Supplemental Table 6. Predictive Margins of Preterm Birth and Low Birthweight from Linear Probability Models Examining Moderating Effects of Race/Ethnicity and Education Level on Relationship between Restrictiveness Index and Adverse Birth Outcomes**

|  | **Preterm Birth** | | | |  | **Low Birthweight** | | | |
| --- | --- | --- | --- | --- | --- | --- | --- | --- | --- |
|  | *-1 SD* | *0 SD* | *+1 SD* | *+2 SD* |  | *-1 SD* | *0 SD* | *+1 SD* | *+2 SD* |
| ***Race/ethnicity (categorical) x Education x Restrictiveness Index (RI)*** | | | | | | | | | |
| AIAN, LT HS grad | 13.2 | 13.0 | 12.7 | 12.5 |  | 7.1 | 7.0 | 6.9 | 6.9 |
| AIAN, HS grad | 11.8 | 11.3 | 10.8 | 10.3 |  | 6.1 | 5.9 | 5.8 | 5.6 |
| AIAN, Some college | 10.9 | 10.9 | 11.0 | 11.0 |  | 6.4 | 6.2 | 6.1 | 5.9 |
| AIAN, College grad | 11.6 | 10.7 | 9.9 | 9.0 |  | 7.8 | 6.8 | 5.8 | 4.9 |
| AAPI, LT HS grad | 12.9 | 13.0 | 13.0 | 13.0 |  | 9.2 | 8.8 | 8.4 | 8.1 |
| AAPI, HS grad | 12.5 | 12.4 | 12.2 | 12.1 |  | 9.0 | 9.0 | 9.0 | 9.0 |
| AAPI, Some college | 12.1 | 12.2 | 12.3 | 12.4 |  | 9.2 | 9.1 | 9.0 | 8.9 |
| AAPI, College grad | 10.7 | 10.3 | 9.8 | 9.4 |  | 8.9 | 8.7 | 8.4 | 8.2 |
| Black, LT HS grad | 15.3 | 15.9 | 16.5 | 17.2 |  | 12.2 | 12.6 | 12.9 | 13.2 |
| Black, HS grad | 15.1 | 15.2 | 15.4 | 15.6 |  | 12.1 | 12.0 | 12.0 | 12.0 |
| Black, Some college | 14.4 | 14.5 | 14.7 | 14.8 |  | 11.4 | 11.4 | 11.4 | 11.4 |
| Black, College grad | 13.5 | 13.3 | 13.2 | 13.1 |  | 10.4 | 10.1 | 9.9 | 9.6 |
| Hispanic/Latinx, LT HS grad | 12.1 | 11.7 | 11.3 | 10.9 |  | 7.4 | 6.8 | 6.3 | 5.8 |
| Hispanic/Latinx, HS grad | 12.2 | 12.0 | 11.8 | 11.6 |  | 7.7 | 7.4 | 7.1 | 6.8 |
| Hispanic/Latinx, Some college | 12.1 | 12.2 | 12.3 | 12.4 |  | 8.0 | 7.7 | 7.4 | 7.1 |
| Hispanic/Latinx, College grad | 11.5 | 11.1 | 10.8 | 10.5 |  | 7.5 | 7.1 | 6.7 | 6.2 |
| White, LT HS grad | 12.6 | 12.3 | 12.1 | 11.8 |  | 9.5 | 9.3 | 9.1 | 8.9 |
| White, HS grad | 11.5 | 11.7 | 11.8 | 11.9 |  | 7.9 | 8.0 | 8.0 | 8.0 |
| White, Some college | 11.3 | 11.2 | 11.1 | 11.1 |  | 7.2 | 7.0 | 6.8 | 6.6 |
| White, College grad | 10.2 | 10.0 | 9.8 | 9.6 |  | 6.4 | 6.1 | 5.7 | 5.4 |
| ***Race/ethnicity (dichotomous) x Education x Restrictiveness Index (RI)*** | | | | | | | | | |
| Black, LT HS grad | 15.3 | 15.9 | 16.5 | 17.2 |  | 12.2 | 12.6 | 12.9 | 13.2 |
| Black, HS grad | 15.1 | 15.2 | 15.4 | 15.6 |  | 12.1 | 12.0 | 12.0 | 12.0 |
| Black, Some college | 14.4 | 14.5 | 14.7 | 14.8 |  | 11.4 | 11.4 | 11.4 | 11.4 |
| Black, College grad | 13.5 | 13.3 | 13.2 | 13.1 |  | 10.4 | 10.1 | 9.9 | 9.6 |
| Non-Black, LT HS grad | 12.2 | 12.0 | 11.8 | 11.6 |  | 7.9 | 7.9 | 7.9 | 7.8 |
| Non-Black, HS grad | 11.8 | 11.8 | 11.8 | 11.8 |  | 7.9 | 7.8 | 7.8 | 7.7 |
| Non-Black, Some college | 11.5 | 11.5 | 11.4 | 11.3 |  | 7.6 | 7.3 | 6.9 | 6.6 |
| Non-Black, College grad | 10.4 | 10.2 | 9.9 | 9.7 |  | 7.0 | 6.5 | 6.0 | 5.4 |

Note: Results are predictive margins of preterm birth and low birthweight for all racial/ethnic-education level subgroups at -1 standard deviation (SD), 0 SD, +1 SD, and +2 SD of the lagged restrictiveness index. Predictive margin estimates were produced from multivariate linear probability models estimating moderating effects of race/ethnicity and education level on the relationship between the standardized lagged restrictiveness index and the probability of preterm birth and low birthweight among all 50 states and Washington, D.C. Final sample size included people not missing any data on race/ethnicity, education level, restrictiveness index, outcomes, and covariates. All models adjust for individual-level sociodemographic characteristics, state-level sociodemographic, economic, and political characteristics, and state and year fixed effects. Standard errors clustered at the state level
