# Supplementary material for: Racial/ethnic and educational inequities in restrictive abortion policy variation and adverse birth outcomes in the United States
Source: BMC Health Serv Res. 2021 Oct 22;21:1139. doi: 10.1186/s12913-021-07165-x (PMC8532280; doi:10.1186/s12913-021-07165-x)
Supplement: Supplementary file 2 — Additional file 2: Supplemental Figure 1. Predictive Margins and Average Marginal Effects of Racial/Ethnic (Categorical) Inequities in Relationship between Restrictiveness Index and Adverse Birth Outcomes. Supplemental Figure 2. Predictive Margins and Average Marginal Effects of Racial/Ethnic (Dichotomous) Inequities in Relationship between Restrictiveness Index and Low Birthweight. Supplemental Figure 3. Predictive Margins and Average Marginal Effects of Educational Inequities in Relationship between Restrictiveness Index and Preterm Birth. Supplemental Figure 4. Predictive Margins and Average Marginal Effects of (Categorical) Racial/Ethnic-Educational Inequities in Relationship between Restrictiveness Index and Preterm Birth. Supplemental Figure 5. Predictive Margins and Average Marginal Effects of (Categorical) Racial/Ethnic-Educational Inequities in Relationship between Restrictiveness Index and Low Birthweight. Supplemental Figure 6. Predictive Margins and Average Marginal Effects of (Dichotomous) Racial/Ethnic-Educational Inequities in Relationship between Restrictiveness Index and Preterm Birth. Supplemental Figure 7. Predictive Margins and Average Marginal Effects of (Dichotomous) Racial/Ethnic-Educational Inequities in Relationship between Restrictiveness Index and Low Birthweight. [file 12913_2021_7165_MOESM2_ESM.docx]

**Supplemental Figure 1. Predictive Margins and Average Marginal Effects of Racial/Ethnic (Categorical) Inequities in Relationship between Restrictiveness Index and Adverse Birth Outcomes**

Note: Results are predictive margins and average marginal effects from multivariate linear probability models estimating the moderating effect of race/ethnicity (categorical) on the relationship between a standardized lagged restrictiveness index and the probability of preterm birth and low birthweight among all 50 states and Washington, D.C. Final sample size included people not missing any data on race/ethnicity, restrictiveness index, outcomes, and covariates. All models adjust for individual-level sociodemographic characteristics, state-level sociodemographic, economic, and political characteristics, and state and year fixed effects. Standard errors clustered at the state level.

**Supplemental Figure 2. Predictive Margins and Average Marginal Effects of Racial/Ethnic (Dichotomous) Inequities in Relationship between Restrictiveness Index and Low Birthweight**

Note: Results are predictive margins and average marginal effects from multivariate linear probability models estimating the moderating effect of race/ethnicity (dichotomous) on the relationship between a standardized lagged restrictiveness index and the probability of low birthweight among all 50 states and Washington, D.C. Final sample size included people not missing any data on race/ethnicity, restrictiveness index, low birthweight, and covariates. All models adjust for individual-level sociodemographic characteristics, state-level sociodemographic, economic, and political characteristics, and state and year fixed effects. Standard errors clustered at the state level.

**Supplemental Figure 3. Predictive Margins and Average Marginal Effects of Educational Inequities in Relationship between Restrictiveness Index and Preterm Birth**

Note: Results are predictive margins and average marginal effects from multivariate linear probability models estimating the moderating effect of education level on the relationship between a standardized lagged restrictiveness index and the probability of preterm birth among all 50 states and Washington, D.C. Final sample size included people not missing any data on education level, restrictiveness index, preterm birth, and covariates. All models adjust for individual-level sociodemographic characteristics, state-level sociodemographic, economic, and political characteristics, and state and year fixed effects. Standard errors clustered at the state level.

**Supplemental Figure 4. Predictive Margins and Average Marginal Effects of (Categorical) Racial/Ethnic-Educational Inequities in Relationship between** **Restrictiveness Index and Preterm Birth**

Note: Results are predictive margins and average marginal effects from multivariate linear probability models estimating the moderating effect of race/ethnicity (categorical) and education level on the relationship between a standardized lagged restrictiveness index and the probability of preterm birth among all 50 states and Washington, D.C. Final sample size included people not missing any data on race/ethnicity, education level, restrictiveness index, preterm birth, and covariates. All models adjust for individual-level sociodemographic characteristics, state-level sociodemographic, economic, and political characteristics, and state and year fixed effects. Standard errors clustered at the state level.

**Supplemental Figure 5. Predictive Margins and Average Marginal Effects of (Categorical) Racial/Ethnic-Educational Inequities in Relationship between Restrictiveness Index and Low Birthweight**

Note: Results are predictive margins and average marginal effects from multivariate linear probability models estimating the moderating effect of race/ethnicity (categorical) and education level on the relationship between a standardized lagged restrictiveness index and the probability of low birthweight among all 50 states and Washington, D.C. Final sample size included people not missing any data on race/ethnicity, education level, restrictiveness index, low birthweight, and covariates. All models adjust for individual-level sociodemographic characteristics, state-level sociodemographic, economic, and political characteristics, and state and year fixed effects. Standard errors clustered at the state level.

**Supplemental Figure 6. Predictive Margins and Average Marginal Effects of (Dichotomous) Racial/Ethnic-Educational Inequities in Relationship between Restrictiveness Index and Preterm Birth**


Note: Results are predictive margins and average marginal effects from multivariate linear probability models estimating the moderating effect of race/ethnicity (dichotomous) and education level on the relationship between a standardized lagged restrictiveness index and the probability of low birthweight among all 50 states and Washington, D.C. Final sample size included people not missing any data on race/ethnicity, education level, restrictiveness index, low birthweight, and covariates. All models adjust for individual-level sociodemographic characteristics, state-level sociodemographic, economic, and political characteristics, and state and year fixed effects. Standard errors clustered at the state level.

**Supplemental Figure 7. Predictive Margins and Average Marginal Effects of (Dichotomous) Racial/Ethnic-Educational Inequities in Relationship between Restrictiveness Index and Low Birthweight**


Note: Results are predictive margins and average marginal effects from multivariate linear probability models estimating the moderating effect of race/ethnicity (dichotomous) and education level on the relationship between a standardized lagged restrictiveness index and the probability of low birthweight among all 50 states and Washington, D.C. Final sample size included people not missing any data on race/ethnicity, education level, restrictiveness index, low birthweight, and covariates. All models adjust for individual-level sociodemographic characteristics, state-level sociodemographic, economic, and political characteristics, and state and year fixed effects. Standard errors clustered at the state level.
